# Updates in non-small cell lung cancer - insights from the 2009 45^th ^annual meeting of the American Society of Clinical Oncology

**DOI:** 10.1186/1756-8722-3-18

**Published:** 2010-05-02

**Authors:** Hamid R Mirshahidi, Chung T Hsueh

**Affiliations:** 1 Division of Medical Oncology and Hematology, Loma Linda University, Loma Linda, CA 92354, USA

## Abstract

We have reviewed the pivotal presentations in non-small cell lung cancer (NSCLC) from the 2009 annual meeting of the American Society of Clinical Oncology. We have discussed the scientific data, the impact on standards of care, and ongoing clinical trials.

In patients with early-stage NSCLC, there is still no data to support the superiority of either neoadjuvant or adjuvant chemotherapy. However, adjuvant cisplatin-based chemotherapy has sustained the survival benefits after median follow-up of more than 9 years. The first-line treatment with inhibitors of epidermal growth factor receptor (EGFR) could be considered for the treatment of EGFR mutated patients with metastatic disease.

Several maintenance studies with cytotoxic or biological agents have also demonstrated promising outcomes. Finally, novel targeted agents such as inhibitors of histone deacetylase and multi-targeted tyrosine kinase inhibitor have shown promising activity in NSCLC treatment.

## Introduction

The 2009 Annual Meeting of the American Society of Clinical Oncology (ASCO) in Florida introduced and highlighted numerous important studies and medical advancements. Among them, the meeting brought forth much data from several key studies in non-small-cell lung cancer (NSCLC). The purpose of this article is to review several important abstracts that were presented in different lung cancer tracts, which may influence the standards of care in the future. With that said, such abstracts include the Neoadjuvant or Adjuvant Chemotherapy in patients with Operable Non-Small Cell Lung Cancer (NATCH) trial and the updated long-term follow-up data from JBR.10 adjuvant chemotherapy study in the early stage disease. This article will also take into account and review the data from trials regarding pemetrexed and erlotinib present in patients with locally advanced disease as the maintenance therapy. Moreover, in advanced NSCLC, there have been new findings from studies that assessed vorinostat efficacy and results from Southwest Oncology Group (SWOG) S0536 evaluating four drug combinations. Lastly, biomarker studies from the Iressa Pan-Asia Study (IPASS) and the first-line Cetuximab in lung cancer (FLEX) trials will be reviewed; such trials managed to reveal predictive factors for inhibitors of epidermal growth factor receptor (EGFR).

The data reviewed in this article were obtained from the results presented in ASCO 2009 annual meeting. Therefore, a possible discordance between these data and the final results published in the papers should be considered.

## I. Chemotherapy in Early-Stage NSCLC

Neoadjuvant chemotherapy studies have shown to improve survival outcomes for patients with stage II or IIIA NSCLC in several randomized studies [1,2]. Data from large randomized clinical trials and pooled analyses have also supported the use of adjuvant platinum-based chemotherapy in patients with completely resected stage II or III NSCLC [3]. A meta-analysis yielded similar overall survival (OS) and disease-free survival (DFS) for patients with resectable lung cancer who received either neoadjuvant or adjuvant chemotherapy [4]. Two presentation in 2009 ASCO meetings have provided additional insights.

### Chemotherapy with carboplatin and paclitaxel provided no additional benefit to surgery in early-stage lung cancer

Felip et al. presented the results from NATCH study, which was a multicenter, phase III study that randomly assigned patients to surgery alone, neoadjuvant chemotherapy followed by surgery or surgery followed by adjuvant chemotherapy [5]. This study enrolled 624 patients with clinical early-stage (stage IA with tumor size > 2 cm, IB, II, or T3N1) resectable NSCLC. Patients on neoadjuvant and adjuvant chemotherapy arms received 3 cycles of carboplatin AUC of 6 and paclitaxel 200 mg/m2 every 3 weeks. The primary end-point was 5-year DFS. After a median follow-up of 43 months, the median DFS was not significantly different among the three arms (28, 32, and 24 months in the surgery, neoadjuvant, and adjuvant arms, respectively). The 5-year DFS rate was also similar among the 3 groups and no significant difference in median OS was observed as well. The rate of resection, types of surgery, and post-operative mortality were similar across treatment groups. Ninety seven percent of patients in neoadjuvant and 66% of patient in the adjuvant chemotherapy group received the planned 3 cycles of chemotherapy. The exploratory analysis of these results showed the patients with clinical stage II and T3N1 disease derived the greatest benefit from preoperative chemotherapy followed by surgery. The data were likely influenced by the facts that nearly 50% of the patients had stage I disease and cisplatin-based chemotherapy regimen was not employed. Cancer Leukemia Group B (CALGB) 9633 also failed to produce a long-term overall survival benefit in patients with stage IB disease who received adjuvant paclitaxel and carboplatin after surgery [6]. Three cycles of neoadjuvant carboplatin and paclitaxel followed by surgery was also studied in SWOG S990. In this study, more than two thirds of patients were classified with earlier stage disease, IB or IIA. This trial closed prematurely in 2004 after several studies demonstrated a significant survival benefit for adjuvant chemotherapy. These results did not quite achieve statistical significance due to early closure, but the study showed a strong trend toward improved progression-free survival (PFS) and OS [7]. Unfortunately, NATCH could not determine the superiority of either neoadjuvant or adjuvant chemotherapy over each other. It is recommended to wait for the results of the ongoing trials in Asia and Europe (ClinicalTrials.gov Identifier: NCT00398385, NCT00321334, and NCT00389688) to resolve this issue. The retrospective analyzing of NATCH is undertaken to define prognostic and predictive molecular markers.

### 2- JBR 10

**Figure 1:**
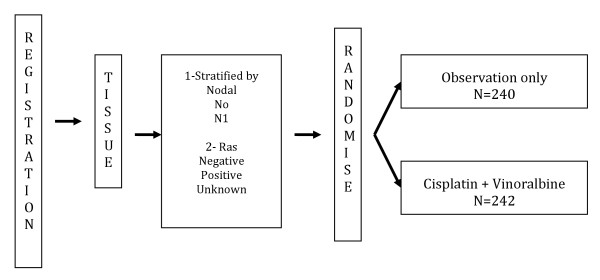
**JBR 10 - Study design of JBR 10 [Reference: **[8]].

Dr. Vincent updated the survival data for JBR.10 with 9 years of median follow up. JBR.10 was a multicenter, randomized controlled trial. Eligible patients included those with completely resected stage IB (T2N0) or II (T1 - T2, N1) NSCLC who were randomized to receive 4 cycles of vinorelbine plus cisplatin or observation within 6 weeks of surgery [8](Figure 1). Baseline characteristics were well-balanced including RAS status. In the updated results, the survival analysis continues to show the benefits from chemotherapy beyond 12 years and suggestive of cure (hazard ratio [HR} 0.78, p = 0.04). In comparison, the updated IALT results with a median follow-up of 7.5 years showed a fading effect of adjuvant chemotherapy on survival. The initial 14% reduction in the risk of death reduced to 9% with adjuvant chemotherapy after 5 year and this difference was no longer statistically significant [9]. The definite benefit appears to be confined to N1 disease. In stage II disease, the median OS was 6.8 years in the chemotherapy arm versus 3.6 years in the observation arm (HR 0.68, p = 0.01). The patients with stage IB did not exhibit a significant benefit (HR 1.03; p = 0.87). However, stage IB patients with tumors greater than 4 cm gained a greater benefit, although this trend was not statistically significant (HR 0.66; p = 0.13). Paclitaxel and carboplatin also failed to produce a long-term overall survival benefit in patients with stage IB disease in CALGB 9633. However, exploratory analysis demonstrated a significant survival difference in favor of adjuvant chemotherapy for patients who had tumors 4 cm in diameter (HR, 0.69; CI, 0.48 to 0.99; *P *= .043) [6]. The RAS mutation status was not significant in COX analysis. Competing risk analysis also showed observation to be associated with significantly higher risk of death from lung cancer (p = 0.02) with no difference in incidences of death from other causes between arms including second malignancy (p = 0.62).

## Metastatic NSCLC

### Maintenance Therapy

The current recommended first-line treatment for patients with advanced stage NCSLC is combination of a platinum-based regimen for 4 to 6 cycles. The prolonged front-line platinum-based chemotherapy does not seem to provide any other additional benefits [10]. Therefore, the current treatment guidelines have recommended waiting until the disease has progressed to initiate second and third-line regimens. Several studies have shown that maintenance chemotherapy may improve PFS in patients achieving disease control after first-line chemotherapy [11]. Maintenance chemotherapy is the extension of chemotherapy duration with additional drugs given after a set course of first-line chemotherapy in patients achieving tumor response including stable disease. Usually maintenance chemotherapy is continued till disease progression or unacceptable toxicity. Three maintenance studies were presented this year.

### Pemetrexed

Dr. Belani gave a presentation on his research and data that supported the notion that patients would greatly benefit from pemetrexed maintenance therapy [12]. The study was a randomized, double-blind, multicenter, phase III study in patients with advanced NSCLC who received four cycles of first-line platinum-doublet chemo. It demonstrated a significant PFS and OS benefit for maintenance pemetrexed and best supportive care (BSC) treatment compare to placebo and BSC (Table 1). In addition, this study confirmed non-squamous histology was predictive of the improved efficacy of pemetrexed maintenance therapy. The administration of pemetrexed in the maintenance setting was fairly well tolerated and was devoid of any cumulative toxicity in the subgroup analysis. The rates of grade 3 and 4 toxicities in the pemetrexed arm were low. Taking that into consideration, the difference in the deterioration of quality of life among pemetrexed and placebo treatments were not reported this year.

**Table 1:** Overall outcome analysis in pemetrexed maintenance study based on histology subgroups

	*Pemetrexed n = 441)*	*Placebo (n = 222)*	*HR (95% CI)*	*P Value*
**Overall Median PFS, months**	4.0	2.0	0.60 (0.49-0.73)	< 0.0001

Median PFS in Nonsquamous cases (n = 481)	4.4	1.8	0.47 (0.37-0.60)	< 0.00001

Median PFS in Squamous cases (n = 182)	2.4	2.5	1.03 (0.77-1.50)	Not Significant

**Overall Median OS, months**	13.4	10.6	0.79 (0.65-0.95)	0.012

Overall Median in Nonsquamous cases (n = 481)	15.5	10.3	0.70 (0.56-0.88)	0.002

Overall Median in Squamous cases (n = 182)	9.9	10.8	1.07 (0.49-0.73)	Not Significant

PFS, progression free survival; OS, overall survival; HR, hazard ratio; Reference: [9]

Another concern of this study was that only 67% of the placebo arm patients received second-line therapy. Meanwhile 51% of the placebo arm patients received third-line and 19% received fourth-line treatment. Only 19% of patients received pemetrexed in the placebo arm. Therefore, it is possible that the survival benefits may have been preserved if more patients on the placebo arm had received pemetrexed. To be clear, this study does not entirely prove that the maintenance strategy is the cause for improved survival. However, this study has reinforced the notion that pemetrexed is an active and effective agent in patients with non-squamous NSCLC.

### Erlotinib

More than 80% of NSCLC overexpress EGFR [13]. Erlotinib is a highly potent EGFR tyrosine-kinase inhibitor (TKI). Erlotinib has been shown to significantly improve the OS and PFS in patients with advanced NSCLC who failed prior platinum-based chemotherapy [14]. In two first-line studies (TRIBUTE and TALENT), combination of erlotinib and platinum-based chemotherapy did not demonstrate improved outcome in advanced NSCLC, compared to chemotherapy alone [15,16].

Sequential erlotinib in unresectable NSCLC (SATURN) trial was a placebo-controlled, randomized, double-blind, phase III study that enrolled 889 patients with advanced NSCLC, and patients were randomized to erlotinib or placebo if their cancer did not progress after at least four cycles of first-line platinum-based chemotherapy [17]. The study met its primary endpoint demonstrating a significantly improved PFS with erlotinib in all comers (HR 0.71; p < 0.0001), and in EGFR-positive subgroup (HR 0.69; p < 0.0001). Additionally, PFS was significantly prolonged with erlotinib regardless of adenocarcinoma or squamous cell carcinoma tumor type (P < 0.0001 and 0.0148, respectively).

Mutation of EGFR was the only marker significantly predictive of differential erlotinib effect (P < 0.001) [18]. Patients exhibiting mutant EGFR tumors had a PFS of about 45 weeks with erlotinib, and 13 weeks with placebo (Table 2). Patients with EGFR wild type tumors had a smaller gain in PFS. This study confirmed that erlotinib is an active and efficacious agent in NSCLC, irrespective of histology. However, the benefits are disproportionate in patients with EGFR mutation.

**Table 2:** Hazard Ratio for Progression Free Survival in biomarkers subgroups in Saturn study.

	HR (95%)	P Value
EGFR IHC positive	0.69 (0.58-0.82)	< .0001

EGFR IHC negative	0.77 (0.51-1.14)	0.1768

*EGFR *positive by FISH	0.68 (0.51-0.90)	0.0068

*EGFR *negative by FISH	0.81 (0.62-1.07)	0.1300

*EGFR *mutation	0.10 (0.04-0.25)	< 0.0001

*EGFR *wild type	0.78 (0.63-0.96)	0.0185

*KRAS *mutation	0.77 (0.50-1.19)	0.2246

*KRAS *wild type	0.70 (0.57-0.87)	0.0009

HR, Hazard Ratio; IHC, immunohistochemistry; FISH, Fluorescence In Situ Hybridization

Reference: [13]

FAST-ACT study, which was presented in 2008 ASCO annual meeting, also tested erlotinib with platinum/gemcitabine chemotherapy with erlotinib continuing as the maintenance therapy [19]. PFS was statistically better in experimental arm (p = 0.0002), but it did not translate in to improved OS.

### Erlotinib and Bevacizumab (ATLAS)

The addition of bevacizumab to first-line carboplatin and paclitaxel chemotherapy conferred a significant improvement in OS, PFS, response rate (RR) in patients with non-squamous-cell carcinoma and a good performance status [20]. The combination of bevacizumab and erlotinib also showed activity in phase II and III NSCLC trials [21,22]. Taking that into consideration, the ATLAS study was conducted to test the hypothesis of maintenance erlotinib in combination with Bevacizumab in patients with advanced-stage [23]. The patients with no progressive disease or significant toxicity were randomized to receive either erlotinib or placebo with Bevacizumab until disease progression after initial therapy. The study included patients with peripheral or extrathoracic squamous cell carcinomas and patients with treated brain metastases. The study met its primary endpoint by demonstrating that patients who received Erlotinib in combination with bevacizumab as maintenance treatment had a median PFS of 4.76 months compared to 3.75 months in the control arm (HR 0.72; p = 0.0012). Adverse events were consistent with previous Bevacizumab or Erlotinib NSCLC studies evaluating the two medicines together. However, the combination arm experienced more adverse events and serious adverse events, including more grade 3-5 toxicities (46.3% vs. 31.55%). The quality of life analysis was not included in this trial. HR for PFS favored erlotinib arm in nearly all patient subgroups regardless of their ethnicity, sex, smoking history, tumor histology, and initial chemotherapy. Data on OS are expected to be announced in early 2010.

## First Line Therapy with Inhibitors of EGFR

### Flex

Dr. O'Byrne presented the retrospective analysis of the data from FLEX trail to identify the molecular and clinical predictors of outcome for cetuximab in NSCLC [24]. In this study, 1125 patients with advanced NSCLC and positive EGFR staining by immunohistochemistry (IHC) were randomized to cisplatin and vinorelbine with or without cetuximab. Patients remained on maintenance therapy until disease progression. A statistically significant difference in OS was found, with improvement from 10.1 months to 11.3 months (p = 0.0441). The RR was also superior in the cetuximab group (29% vs. 36%, p = 0.012). However, PFS was identical at 4.8 months in each group. In a subgroup analysis, patients with squamous cell histology retained survival benefit. Subgroup analysis of Asian patients included in the study (n = 121) did not show an improvement in survival with the addition of cetuximab (median OS, 17.6 months for chemotherapy plus cetuximab vs. 20.4 months for chemotherapy alone, not statistically significant). However, on disease progression, the Asian subgroup that received cetuximab also received fewer EGFR TKIs (50% vs. 73%). The lack of this may have had a negative effect on their outcomes [25].

Patient selection with positive IHC of EGFR might not be the right selection criteria in this study. Most cetuximab studies have not clearly shown an association between EGFR expression and response. However, the results from SWOG 0342 study have shown that the amplification of EGFR gene copy number, determined by fluorescent in situ hybridization (FISH), might predict an improved survival for EGFR TKI therapy [26]. Among FISH-negative patients, median OS was 10.6 months with chemotherapy and cetuximab compared to 10.0 months with chemotherapy alone (HR, 0.91). In FISH-positive patients, median OS was 11.6 months in the cetuximab arm while it was 9.9 months in the control arm (HR, 0.85). Similarly, PFS and RR by FISH status failed to indicate response to cetuximab therapy. KRAS mutation status did not affect OS, PFS, or RR in either subgroup. The KRAS and EGFR-biomarker data are congruent with those from the smaller BMS-099 trial, in which cetuximab was added to a taxane and carboplatin in the first-line treatment of NSCLC [27].

The most important finding of the analysis was the first-cycle rash, which might help to identify patients with improved survival with cetuximab. The median overall for survival was 15.0 months in patients that developed an acnelike rash of any grade within 21 days of treatment with cetuximab and chemotherapy in comparison to 8.8 months for those without a rash after cetuximab treatment (HR 0.63; p < 0.001). The survival was 10.3 months in the chemotherapy-alone arm. The median OS was 15.0 months in 290 patients with a grade 1-3 rash and 14.7 months in 120 patients with a grade 2-3 rash. It might indicate that the development of a rash is important predictive factor than the specific grade of the rash. The data depicted the OS to be far more superior when cetuximab was added to the standard first-line chemotherapy regardless of histology or KRAS mutation and EGFR gene copy number status. An important question to consider is: if the first-cycle rash is a predictive clinical biomarker, should we continue with cetuximab in patients with no signs of rashes? Overall, the findings suggest that the optimal selection strategy for treatment with cetuximab remains to be defined.

#### SWOG 0536

SWOG 0536 was a phase II study that evaluated the effectiveness and safety of utilizing combinations of bevacizumab, paclitaxel, carboplatin, and cetuximab in patients with advanced-stage NSCLC [28]. Bevacizumab and cetuximab were continued after 6 cycles of chemotherapy till progression of disease. In this study, 104 patients with newly diagnosed stage IIIB or IV NSCLC were treated. Overall, this 4-drug combination was shown to be active with favorable efficacy. An analysis of molecular biomarkers showed that neither KRAS nor EGFR mutations were predictive of outcomes. In addition, although there was a trend toward improved tumor response and disease control rate in patients with EGFR-positive tumors by FISH, no significant differences were noted in PFS or OS. Further analysis of other translational studies such as, EGFR status by FISH, cytokine and angiogenic factor profiling, and proteomics are still ongoing.

The SWOG 0536 study met its primary tolerability endpoint. This combination may also have an additive rather than a synergistic effect. However, the synergistic benefit may be seen in a subset of patients. The positive results of this trial warrant the continued investigation of this 4-drug combination in the phase III SWOG 0819. This study, with a planned enrollment of 1,545 patients, will compare initial therapy (paclitaxel/carboplatin plus bevacizumab with or without cetuximab) followed by maintenance therapy (bevacizumab with or without cetuximab). The primary endpoints are OS in entire study population and PFS in EGFR FISH positive patients.

## Ipass

EGFR TKIs have comparable clinical efficiency with the best supportive care or standard chemotherapy as second-line or third-line therapy for advanced non-small-cell lung cancer [14,29]. They are most effective in women, patients who have never smoked, patients with adenocarcinoma, and patients of Asian origin [30]. These populations have also relatively high incidence somatic mutations in tyrosine kinase domain of EGFR gene including base-pair deletion at exon 19 or a point mutation at exon 21 [31]. The studies of first-line therapy with these agents showed objective RR of 54.8 to 81.6% and PFS of 9.7 to 13.3 months among patients with these mutations [31,32]. Therefore, the IPASS study was conducted to assess the efficacy, safety and tolerability of gefitinib compared to carboplatin and paclitaxel as first-line treatment in a clinically selected population of patients from Asia [33].

The results of planned exploratory analysis to predict the efficacy of treatment based on EGFR mutation, EGFR gene copy number, and EGFR protein expression were presented in 2009 ASCO meeting [34]. One thousand two hundred seventeen patients in Asia with advanced NSCLC whose tumors were of adenocarcinoma histology and who had either never smoked, or were former light smokers were randomized in a 1:1 ratio to receive 250 mg gefitinib per day till progression of disease or paclitaxel (200 mg/m2) and carboplatin (AUC 5.0 or 6.0) every 3 weeks for up to 6 cycles. Biomarker status and incidence of specific identified EGFR mutations were well balanced between treatment arms. The study met its primary objective and it showed the statistically significant improved PFS in gefitinib subgroup, with hazard ratio of 0.74 (p < 0.001). PFS favored chemotherapy for the first 6 months than gefitinib, likely driven by EGFR mutation statue or continuation of gefitinib as maintenance therapy. The PFS and RR were similar in all subgroups in biomarker analysis.

EGFR mutation was the significant positive predictive factor for PFS in patients who received gefitinib (p < 0.0001). Gefitinib improved PFS in patients with EGFR mutation whereas it reduced PFS in patients without EGFR mutation. The PFS in overall IPASS population and in patients with unknown EGFR mutation who received Gefitinb was similar. The difference in OS was not statistically significant due to small number of events and significant number (39%) of patients in both arms received post-study Carbopaltin/Paclitaxel and gefitinb. However, the trend was toward superior OS with gefitinib among patients with EGFR mutation and with carboplatin and paclitaxel in patients without EGFR mutation (table 3).

**Table 3:** Progression Free Survival and 2-year OS in IPASS Study based on EGFR mutation

Outcome	Gefitinib	Carboplatin+Paclitaxel	HR (95% CI)	P Value
***Median PFS, months***				

EGFR mutation	9.5	6.3	0.48 (0.36-0.64)	< 0.0001

No EGFR mutation	1.5	5.5	2.85 (2.05-3.98)	< 0.0001

***2-year OS, %***				

EGFR mutation	71.2	66.7	0.78 (0.50-1.20)	Not Significant

No EGFR mutation	42.9	50.6	1.38 (0.92-2.09)	Not Significant

PFS, progression free survival; OS, overall survival; HR, hazard ratio; EGFR, epidermal growth factor receptor.

Reference: [30]

EGFR gene copy number was furthermore predictive factor for PFS in patients treated with gefitinib (p = 0.0437). Gefitinib improved PFS in patients with high EGFR copy number significantly (HR 0.66; p = 0.005). The improvement in PFS possibly was driven by overlap with positive mutation status. Patients with both mutation and high copy number of EGFR showed substantially extended PFS with Gefitinib (HR 0.48). In contrast, PFS was significantly shorter mutation-negative patients with high copy number of EGFR (HR 3.85). Gefitinb also improved RR in patients with mutated EGFR, whereas, carboplatin plus paclitaxel improved RR in Mutation-negative patients. (Table 4).

**Table 4:** Overall Response Rate in IPASS Study, based on EGFR mutation, copy, and expression

ORR %	Gefitinib	Carboplatin+Paclitaxel	HR (95% CI)	P Value
EGFR mutation	71.2	47.3	2.75 (1.65-4.60)	0.0001

No EGFR mutation	1.1	23.5	0.04 (0.01-0.27)	0.0013

High EGFR copy number	58.9	44.8	1.79 (1.08-2.96)	.0243

Low EGFR copy number	22.2	26.3	0.80 (0.38-1.68)	0.5580

EGFR protein expression	51.5	41.8	1.49 (0.92-2.42)	0.1093

No EGFR protein Expression	34.0	26.1	1.44 (0.60-3.47)	0.4146

HR, hazard ratio; EGFR, epidermal growth factor receptor.

Reference: [30]

One hundred thirty two patients were positive for EGFR mutation, gene copy number, and protein expression in this study. Only 31 patients were negative for these three factors. The observed degree of overlap among them was higher than previous gefitinib studies. These results cannot be extrapolated directly to a North American population, since there was high degree of overlap among this highly specific patient population. Therefore, never smokers, female, or Asians and patients with adenocarcinoma should be screened for EGFR mutation. EGFR inhibitor therapy should be considered a standard approach for first-line therapy in patients with EGFR mutation. Chemotherapy would be the treatment of choice for patients with unknown EGFR status.

## Novel Agents

### Vandetanib

Vandetanib is an orally bioavailable, anilquinazoline derivative, multi-targeted TKI targeting vascular endothelial growth factor receptor (VEGFR)-2, EGFR, and RET tyrosine kinases [35]. This compound inhibits two key pathways in tumor growth: VEGFR-dependent tumor angiogenesis and EGFR-dependent tumor cell proliferation and survival. Vandetanib was efficacious and well tolerated in patients with advanced solid tumors have demonstrated that the once-daily oral administration of this multi-targeted agent at 300 mg daily was well tolerated and recommended for phase II studies [36]. The subsequent phase II randomized trial involving patients with recurrent NSCLC showed the addition of vandetanib to docetaxel significantly improved PFS [37]. The data from three different studies with vandetanib in NSCLC treatment was presented in Orlando. There was no targeted selection in either of these studies.

### Zodiac

ZODIAC was a randomized, double-blinded, placebo-controlled phase III study evaluating the combination of vandetanib with docetaxel in comparison to just docetaxel in 1,391 patients with advanced NSCLC and previously treated with one prior therapy [38]. All tumor histology, treated brain metastases and previous bevacizumab exposure was permitted. The study met its primary endpoint when it demonstrated that the addition of vandetanib with docetaxel resulted in a statistically significant improvement of PFS (4.0 vs. 3.2 months in all patient populations, including females (Table 5). This result was fairly modest, although, the PFS was statistically significant. HR for PFS generally favored vandetanib arm across clinical subgroups defined by sex, race, smoking status, previous bevacizumab, disease stage, histology, and number of affected organs. The HR for PFS also favored vandetanib arm regardless of baseline tumor and blood biomarker subgroups, with few exceptions in cases with negative EGFR gene amplification and positive KRAS mutation. Retrospective analysis suggested baseline serum VEGFR2 level might serve as a predictive biomarker.

**Table 5:** PFS and OS in Zodiac, Zeal, and Zest studies.

TRIAL	Zodiac		Zeal		Zest	
**Treatment**	**Vandetanib+Docetaxel**	**Placebo+Docetaxel**	**Vandetanib+Pemetrexed**	**Placebo+Pemetrexed**	**Vandetanib**	**Erlotinib**

Median PFS	4.0 Months	3.2 Months	17.6 Weeks	11.9 Weeks	11.3 Weeks	8.9 Weeks

HR	0.79	0.91	0.86	0.86	0.98	1.01

P-Value	< 0.001	0.196	0.108	0.22	0.72	0.83

Median OS Months	10.6	10.0	10.5	9.2	6.9	7.8

PFS, progression free survival; OS, overall survival; HR, hazard ratio;

Reference: [34-36]

The improved OS was not statistically significant but it was in favor of adding vandetanib to docetaxel (10.6 vs. 10.0 months). The HR for OS according to patients, clinical subgroups, tumor, and blood marker subgroups was near 1.0, with exception of EGFR gene amplification (HR of 0.48). The final OS result will be available in the future. The most common adverse events in the experimental arm were rashes, diarrhea, neutropenia, and hypertension. The vandetanib arm did not show any increase in hemoptysis or thrombotic events. The incidence of QTc prolongation was <2%. Unfortunately, the 3-week improvement in PFS may not be clinically meaningful. These data are not likely to change practice until the results of the placebo-controlled ZEPHYR trial (clinicaltrials.gov identifier: NCT00404924) is reported in the first half of 2010. ZEPHYR trial is a direct comparison of vandetanib to placebo in patients previously treated with anti-EGFR therapy.

### Zeal

ZEAL was a randomized, double-blinded, placebo-controlled phase III study evaluating vandetanib and pemetrexed in comparison to just pemetrexed [39]. The study enrolled 534 patients previously treated with one prior first-line therapy for advanced NSCLC. The combination of vandetanib and pemetrexed did show a positive trend in the prolongation of PFS compared to pemetrexed alone (17.6 vs. 11.9 weeks). However, the addition of vandetanib to pemetrexed did not benefit patients with squamous cell carcinoma. The findings from ZEAL were in agreement with ZODIAC results although the primary endpoint did not reach statistical significance in the ZEAL study. The large sample size in ZODIAC may explain the significant improvement in PFS even though the PFS was almost the same in both studies. Evaluation of secondary endpoints in the ZODIAC and ZEAL studies also showed that the addition of vandetanib to chemotherapy significantly improved RR (p < 0.001). These studies also showed that adding vandetanib to chemotherapy resulted in a significantly longer time for the deterioration of disease related symptoms.

### Zest

ZEST was a randomized, double-blinded, phase III study evaluating the efficacy of vandetanib 300 mg versus erlotinib 150 mg [40]. The study enrolled 1240 patients with locally advanced or metastatic NSCLC after failure of at least one line of chemotherapy. While the primary objective of demonstrating a statistically significant prolongation of PFS for vandetanib was not met in this study, in a pre-planned non-inferiority analysis, vandetanib was shown to have similar efficacy to erlotinib for PFS and OS. The RR and symptom control were also similar for both treatments.

The result of ZEST, ZEAL, and ZODIAC trials may not justify the use of vandetanib alone or in combination with chemotherapy in unselected patients at this time. The benefit of adding VEGF inhibition to EGFR inhibition remains unproven. Predictive biomarkers for anti-angiogenesis therapy are needed to select the optimal patient population.

### Histone Deacetylase Inhibitor

Histone deacetylases (HDAC) are a family of enzymes that play an important role in the regulation of gene transcription. Aberrant transcriptional activation and repression mediated by histone acetyltransferases and HDACs occurs in various malignancies. Increase in histone acetylation transforms DNA to more open configuration. Non-transcriptional effects of HDAC also increases acetylation of nonhistone proteins such as hypoxia inducible factor-1 alpha, heat shock protein 90, and α-tubulin to promote cell death, inhibition of angiogenesis, induction of cellular differentiation, modulation of immune gene expression [41].

Vorinostat is a small molecule that inhibits HDAC activity. Vorinostat not only promotes the induction of genes, but also causes the repression of several genes, such as thymidylate synthetase and vascular endothelial growth factor receptor. Inhibition of HDAC activity by vorinostat also results in an increase of acetylated non-histone proteins, such as cytoskeletal proteins, molecular chaperones, and nuclear import factors. Vorinostat is already approved for treatment of cuteneous T-cell lymphoma. Unfortunately, this agent is not active as single-agent in treatment of NSCLC [42]. However, it showed synergistic effect with taxanes due to inhibition of tubulin deacetylator HDAC and Platinum drugs by increasing DNA fragmentation in preclinical and phase I studies [43].

The randomized, double-blind, placebo-controlled phase II study of carboplatin and paclitaxel with or without vorinostat was presented [44]. In this study no crossover between treatment arms and no maintenance therapy were permitted. There was no patient selection related to the target agent. The results of this study showed statistically significant improved tumor RR with vorinostat (34%) compare to (12.5%) in placebo (p = 0.021), suggesting vorinostat enhanced the efficacy of chemotherapy. The RR was improved in both squamous and non-squamous histology. The study was not powered to adequately determine PFS and OS, however, the trend for both favored vorinostat (6.0 vs. 4.1 and 13.0 vs. 9.7 months, respectively.). The divergence of OS curves occurred late, possible due to a subset of patient who did benefit from vorinostat or failure of randomization to adequately balance arms. Major toxicities, such as cytopenias, fatigue, and nausea/vomiting, were more frequent with vorinostat than placebo. Only thrombocytopenia was statistically more common in experimental arm. More treatment-related deaths also occurred in the vorinostat than placebo arm (3% vs. 0%). Therefore, the optimizing of dose and schedule of vorinostat is required to improve the tolerability of the combination. This study suggests that targeting different pathways other than EGFR and angiogenesis signaling pathways may play an important role in the treatment of NSCLC.

## Conclusion

As the conclusion, the results from JBR.10 are reassuring and show no long-term, non-lung cancer-related deaths and the long-term positive results could be due to type of chemotherapy regimen or biologic characteristic of patients and the tumors. However, offering adjuvant chemotherapy to stage IB patient still depends on the individual cases. Unfortunately, NATCH did not show any benefit of perioperative chemotherapy in addition to surgery.

There is no gold standard and consensus between oncologists regarding maintenance therapy. Some patients may benefit from maintenance therapy, however, some will also be overtreated. We should also consider many patients still enjoy a treatment holiday. Therefore, these trials may actually indicate that exposure to more active agents improves outcomes rather than validating the concept of maintenance and selection of appropriate treatment should be considered on an individual patient basis.

In terms of biomarkers, we still do have conflicting results except the documented importance of EFGR mutation. The routine use of EGFR FISH or IHC testing as well as KRAS testing for making decisions in the first-line treatment setting cannot be recommended at this time. Lastly, there is hope for improving outcomes in the second-line setting given the positive data from the ZODIAC trial. The important finding in this trial has demonstrated the improvement of PFS and RR from vandetanib translated into the clinically meaningful delay in symptom progression.

## Competing interests

The authors declare that they have no competing interests.

## Authors' contributions

Both authors participated in drafting and editing the manuscript. Both authors read and approved the final manuscript.
